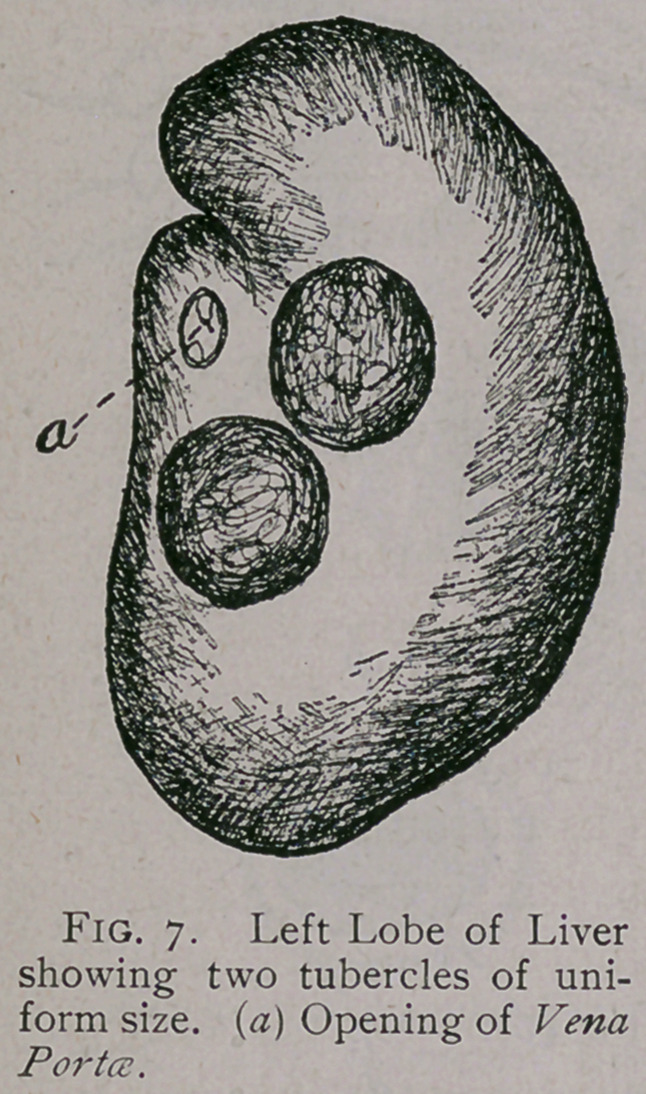# Investigation upon the Diseases of Rodents

**Published:** 1892-06

**Authors:** S. E. Weber

**Affiliations:** Assistant State Veterinarian, Penn.


### INVESTIGATION UPON THE DISEASES OF RODENTS.

### THEIR RELATION WITH COMPARATIVE PATHOLOGY AND PUBLIC HEALTH.

By S. E. WEBER, V. S., Assistant State Veterinarian, Penn.

I have for the last seven years been carrying on a series of post-mortem examinations upon domestic animals in order to study Comparative Pathology, and have had especial advantages at the Lamparter Glue Works, just outside of Lancaster, Pa , where dead animals, brought from all over the county, are worked up.

Observing from time to time the numerous rats in the neighborhood and around the works, and the fact that the workmen of the establishment frequently told me that many of them were taken sick and died, aroused my mind to know what diseases rodent flesh was heir to. It thus occurred to me to investigate the diseases of this constant messmate (for such it might be designated) and companion of the domestic circles of man, and try to discover what bearing might be brought about in relation with Comparative Pathology and Public Health. Not having the facilities at hand or sufficient time as a practitioner of veterinary medicine to properly carry on such an investigation, the matter was postponed from time to time and limited to occasional examinations as time afforded, and it was therefore not until April, 1891, that the work was really commenced.

On the second of April, of that year, while holding some post-mortems upon horses and cattle at the place mentioned, my attention was more particularly drawn to a sick rat crawling about the yard of the establishment, when I determined to begin work immediately. This proved to be one of the most interesting cases of my early collection. I obtained a number for the purpose of studying them closely, but as this was a sick subject it may be of some interest to mention a few of its more prominent external symptoms This rat came crawling into the yard apparently heedless of any surrounding danger to itself. It did not try to get out of the way of the dogs, and could easily be picked up by the hand without offering the least resistance or trying to bite. Some of the other objective symptoms were : General debility, great emaciation of the body, arched back, staring coat, haggard and distressed expression in the face, loss of appetite, mucous membrane very pale, dysponea, cough, discharge from the nostrils. And in ten hours afterward it was found dead.

The next case was one that was seen to come out of its hole every day, near where the carcasses were unloaded and skinned, to feed from a fresh subject that had come in. This one was more active, but not enough so to get away, and so was caught for the purpose of marking, and it was then let go again to have its own freedom It was then noticed to appear almost daily for its rations,

FIG. 1.

NOTE.—Natural size just as drawn from nature after spreading the specimen on a card. The same is true of all the figures in this article.

FIG. 1. The Lungs of the first case noted. The Left Lung was apparently healthy, but the posterior and middle lobes were a mass of tubercles, the larger containing yellow matter that had not yet become cheesy. The anterior lobe was but slightly affected on the lower side.

and the same symptoms as mentioned above were shown. In fourteen days afterward I found it dead in the boiling department of the works. The post-mortem appearance of the lungs from these two cases just cited is represented by Figs. 1 and 2.

I will here state that the reason for giving this rat the freedom of her natural habitat, was that they have been found to die very soon after close confinement. Furthermore, they demand fresh meat every day, and if in close confinement the meat gets stale or putrid, and if they are pressed for hunger they do not hesitate to practice cannibalism by a general fight, the stronger devouring the weaker. They feed on the muscles of the neck and head of their own kind, and sometimes if the victim proves to be a young and tender rat, leave nothing of its body but the skin.

I have thus far made upward of one thousand post-mortems upon rats from the above-named place, and collected a large number from other sources, and in them I find such a rich field of pathology that a description will take more time than was first set aside for this inquiry.

Some one has said "rats really render good service to the promotion of public health, acting as scavengers in devouring animal and vegetable substances, the putrefaction of which would otherwise be productive of pestilence. Such, indeed, seems to be the great use of the rat in the economy of nature ; and it is perhaps worthy of notice that the visits of the plague to Western Europe and to Britain have ceased from the very time when rats became plentiful."

As regards this statement, I can only say that the facts already known would seem to be to the contrary. It might be that if "animal and vegetable substances" should be allowed to putrefy, their various constituent parts would return sooner to their source, and would not be so productive of harm as if taken up in some animal economy which would act as a medium and still keep it on its road of development, and along it would distribute detachments of its great army of havoc (like Johnstown floods), at last entering into its original and proper position of nature once more. May not the rat be a vehicle of transmission of disease in face of the above statement from Chamber's Encyclopedia? Did those rats that "became plentiful" in Western Europe and Britain take the plague and keep it? This would seem unreasonable at once, even from the rat, as all things must go onward, continually changing and being reproduced. I will, however, try and discover if the rat's stomach is a prison or furnace for pathogenic-micro-organisms, or if it is the same as that of any other animal of the mammalian species, which would seem to be the case from the facts known. I do not believe that this is the "great use of the rat in the economy of nature."

It is from this fact, that while coming up the steps of the higher organisms of the Protoplasmatic world I stopped in that department devoted by nature to rodents. Stopping on this step, I thought there must be a field for something, knowing that all things must come from below up by gradation. I thus stopped here to see what could be learned, as I knew of no one who had ever made it a special study to look into the diseases of this animal with special reference to Comparative Pathology and its relation to public health. Where can you go to-day, may it be barnyard, kitchen, or palace, without always finding present that ever constant companion of man? The rat being despised by every one, the main object is to destroy it; but remember it has an equal right with all creatures, even from the cell to a gnat or whale; and we should, therefore, not depise any of the links of that endless chain, but see what relation it bears to the highest order under our heading. Allow me here to add a few lines from Cowper:

> "I would not enter on my list of friends,
> Though graced with polished manners and fine sense,
> Yet wanting sensibility, the man
> Who *needlessly* sets foot upon a worm."

Considering the rat psychologically, we find in her a very intelligent and shrewd animal. I will later give some of her feats in this direction when I treat her zoölogically.

### TUBERCULOSIS.

This most cruel and deceptive monster of all the scourges amongst the various diseases of mankind will be given first place for a few words of introduction to these studies. This disease is very common in rats surrounding the glue works, and bears with it all the characteristic pathological organic lesions as is found in the lower order of animals. The first two cases upon which post-mortems were made showed the lungs to be badly affected with this disease, as will be seen by Figs. 1 and 2.

The general condition of the body, such as emaciation and pale appearance of the flesh portrays that of a well-marked case.

I have found it in various forms and stages in the lungs, which are, occasionally, such a mass of disease that it seems miraculous that the animal could have lived. Lesions have been found in the bowels, generally appearing in small groups of tubercles, spreading over the small intestine in numbers from one, two, three, five, eight, thirteen, or twice that number. * The colon and cæcum are not so frequently affected, but when this is the case it is generally more extensively diseased than the other part of the bowels. The mesentery glands are often affected, and those between the colon and cæcum more so than the rest of the chain. The lymphatic glands, in connection with the forearm and neck, also the mammary glands, sub-maxillary glands, liver and spleen, all have their share of affection in various degrees. I will, however, mention that the latter has been found very rarely so affected.

After the investigation was systematically begun I soon found that tuberculosis was the most common disease. The examinations made within the last year show the percentage of well marked

* I will here state that similar post-mortem lesions were found in the bowels of a number of dogs and cats, the size varying with the size of the animal.

cases to be 80 and I have no hesitation in saying that if closer observations were taken it would be still higher. For the present purpose we will mention a few morbid anatomical lesions as represented by drawings of the various organs found affected.

Before entering into the morbid anatomy of the alimentary canal, it should be essentially necessary to first consider the details of the anatomy of this part of the rat's economy, so as to have a clear understanding of the parts in question. As these lines are that of compulsion on my part, circumstances will not permit at present for such detail of anatomy which will be mentioned at the close

FIG. 3.

FIG. 3. Portion of Jejunum. (*a*) shows the regular position of the little tubercles, which is nearly always the case when they are placed in numbers from 5 to 7.

of this article. I will, consequently, mention a few pathological lesions of this part for the present, and treat this subject more fully when I get under the head of this work which will be devoted to pathology. The workmen of the factory frequently noticed rats to be sick about the place and linger until they died. Taking full advantage of this opportunity I was enabled to make myself fully acquainted with the leading post-mortem features of this most interesting disease.

In the first place we will mention some of the appearances which are nearly always found in a typical case of this disease in the bowels. Upon opening the abdominal wall we sometimes observe a small quantity of fluid escape from the peritoneum, while a considerable effusion is sometimes present, and in the latter stages the bowels will adhere together, the adhesions further attaching the whole mass to the abdominal wall. Adhesions of the colon and cæcum are also of common occurrence, and they generally follow in the latter stages when the mesenteric glands of this part of the bowels have been affected, and sometimes small portions of the bowels become matted with it, and then form a common adhesion to the abdominal wall of that part.

There are frequently small groups of tubercles found beneath the serous coat of the small intestine. They are also occasionally found on the colon and cæcum, more rarely in the latter, but when found in greater degree they generally line the whole colon and now and then spread over the cæcum. These little groups of tubercles when not found in a general distribution as just mentioned, are found to consist in numbers from four to twenty, sometimes placed in a most beautiful symmetrical position as represented by fig. 3, which is a portion of the jejunum showing the arrangement of groups in various numbers, which are very seldom found in any other position, but on the greater curvature of the bowel.

These bodies in the first stages merely appear as little round rings or outlines, and from this fact are very easily overlooked, as I found in my earliest post-mortems. They first contain a light colored serum (the interspace being of a greyish hue), and later they turn to a light amber, and subsequently to a grey or yellowish-grey color. Small groups that are closely placed toward the latter stage sometimes form together and project from the bowel as one solid mass, as shown by *b* in Fig. 3. I am not at present able to state if this is the natural process of development of these little colonies, but I have not yet observed the same projection in any of the earlier stages. It therefore may be said that the serous side of the bowel containing this form of lesion is generally smooth and regular. Upon section of the bowel it will be found that the mucous membrane presents a marked difference from the peritoneal aspect, in this, that we now see small protuberances corresponding to the little smooth bodies on the other side.

When the wall of the intestine is pressed upon, there issues forth a grey or grayish-yellow material, always rupturing on the mucous side, unless it is one that is well advanced and projects on the serous side, when it will sometimes rupture on that side. There are also tubercles found on the inside of the bowel, in both small and large intestines (oftener in the latter than in the former) to such an extent as to cause mechanical obstruction from intussusception, induced by tubercular degeneration. Fig. 4. This is more the case when we find an annular tubercular mass in this part of the alimentary canel. We will note as an example that the above form is represented in the cæcum as well as possible by drawing *a* in Fig. 5.

The nodules on the outside of the gut, as shown in Fig. 6 contain a thick fibrous wall that is well supplied with capillaries and are of pale color, and when cut open contain a soft greyish matter sometimes the consistence of cream and again that of a cheesy substance of a yellowish color.

FIG. 4.

FIG. 4. The Cæcum and portion of Colon. (*a*) Cæcum cut open to show tubercular condition on inside. (*b*) Section of Colon showing the same conditions on inside.

*Liver.*—Tuberculosis of the liver is not of frequent occurrence, and when present the tubercles vary in size from a very minute point to that of a common hazel nut. Fig. 7. The external appearance is that of a slight protuberance, yellowish in color, and in strong contrast to the liver. Upon incision there will be found to exude a thick yellowish matter, if it is not a case sufficiently advanced to have become inspissated. In the early stages it is sometimes dfficult to distinguish between the cysticercus of the *tænia crossicolis* of the cat.

I show a few cuts of the most interesting of all the anatomical lesions of this disease, as this is apparently the most desirable field for this affection in the rat. With this hasty prelude of my work I cannot take up the various classes of pathological alterations which I have found, in detail, but will endeavor to show later on how fully my investigations substantiate the importance of my work. Time will, therefore, not allow me to take up the different forms of Lung lesions. The most remarkable of all the wonders in my investigations is the durability of this animal in harboring disease of this kind, as I have observed quite a number of lungs as badly affected as Fig. 1 and 2 when the rat was apparently not sick. I believe this animal can bear more disease than any other animal I know of.

I have (among other diseases) found Pleurisy (with adhesions) and Pneumonia. Diseases of the eye, such as Opthalmia, Cataract, Fungus Hæmatodes, and so forth. Various forms of skin diseases, the most important of which is Favus or *Tinea favosa*, a parasitic

FIG. 5.

FIG. 5. (*a*) Annular Constriction of Cæcum. (*b*) Tubercular Nodule on the Colon. (*c c*) Tubercular Enlargement of the Mesenteric Glands between *Colon* and *Cæcum*. (*d*) Ileum cut off.

cutaneous disease, the existence of which is known in the domestic animals, and man, which may be directly transmitted by the rat, giving it to the cat, and the cat in return to the fondling child, and others.

Diseases of the large joints, probably of Tubercular origin, are found. They are generally swollen to a great degree and very hard, and when cut into, will be found to contain small cavities surrounded by a fibrous wall, and containing yellow matter. Sometimes the joint is found open, and when pressed upon, a large quantity of pus will escape. In the latter stages the bones will be found affected. Calcareous deposits in the liver are of very common occurence, and sometimes of enormous extent, some forms of Tumors have been observed of which I will mention Epithelioma. Several cases presented a formidable looking aspect, as the whole side of the face, taking in the eye, part of the nose, and part of the ear, were found eaten away, exposing the whole side of the jaw. The same has been found on other parts of the body.

Parasites found { Ectozoa. Entozoa. Hæmatozoa. Entozoa { Cestoida. Nematoda.

FIG. 6.

NOTE.—This individual had three groups of Tubercles in the small bowels besides having a portion of the left Lung affected.

FIG. 6. (*a a*) Tubercular Nodules. (*b b*) Fibrous wall cut open to show cavity. (*c c*) Fibrous Wall. (*d*) Vascular mesh surrounding nodule. (*e*) Two enlarged Mesenteric Glands. (*f*) Lymphatic Chain somewhat enlarged.

I have (among other parasites) found *Tænia armata* of the liver, which is the *Cysticercus fasciolaris* Rud; this and Pentastomata to my present knowledge are the only parasites described in the rat. I have found the former parasite to be very common. They appear in numbers from one, two, three, to one hundred or more individual Hydatids. Sometimes one cyst will contain more than one worm.

Occasionally they are found loose in the abdominal cavity, and more rarely in the Pleural cavity.

Among the tænia of the bowels I have found apparently three species, one of which is a very nice specimen of from twenty to thirty-five inches long in its adult form. Its mature segment contains a very nice six-hooked embryo, which at once sets forth the most beautiful example of embryonic life by its rythmical movements and other phenomena. It has been observed for hours at a a time, and every point of the microscopical field speaks volumes of that wonderful, stupendous, and almost inconceivable immensity of the great handiwork of nature's living or protoplasmic world. The frequency of movement has been observed ordinarily to be that of about eight to twelve times per minute, but if irritated by heat its action will be increased to three times that number.

FIG. 7. Left Lobe of Liver showing two tubercles of uniform size. (*a*) Opening of *Vena Portæ*.

*Nematodes.*—Of this class there have been found one species in the bowels. Filaria, and several species of Hæmatozoa, all of which parasites will be described as time affords.

I cannot at present go into the other diseases under consideration, but shall in the future, as time permits, publish a series of articles in THE JOURNAL OF COMPARATIVE MEDICINE AND VETERINARY ARCHIVES, taking up first the anatomy of the rat, and further on its zoology and pathology.

It was not my intention to make public my researches and discoveries until I could publish the anatomy and carefully rearrange and compile the necessary manuscript and cuts to scientifically discuss such an important subject, but owing to a newspaper reporter (in the early part of last March) accidentally surprising me and publishing a report of my premature work, starting other investigators in the same field, thereby forcing me to make the above hurried and incomplete statement, although reluctantly.

---

NOTE.—On page 376, last clause of 2d paragraph, in place of the words "a description," the word *it* should appear, which will convey the meaning intended.

## Figures and Tables

**Fig. 1. f1:**
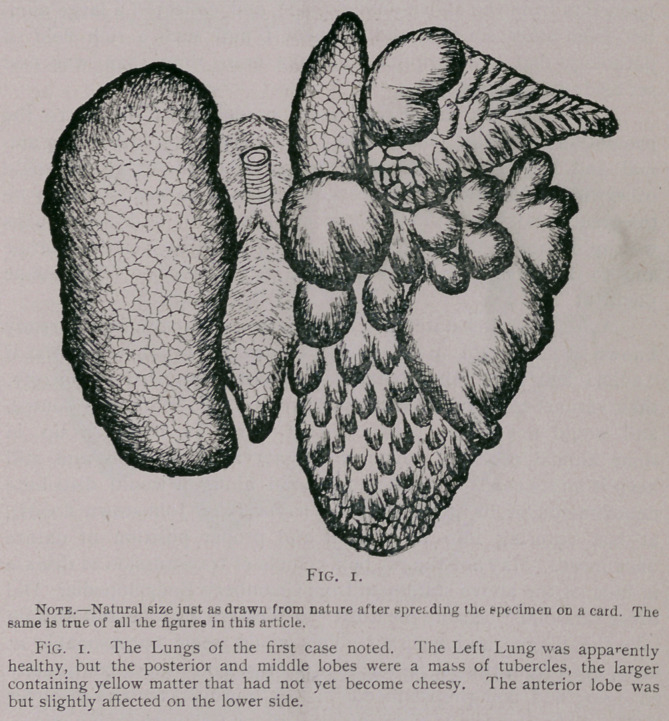


**Fig. 2. f2:**
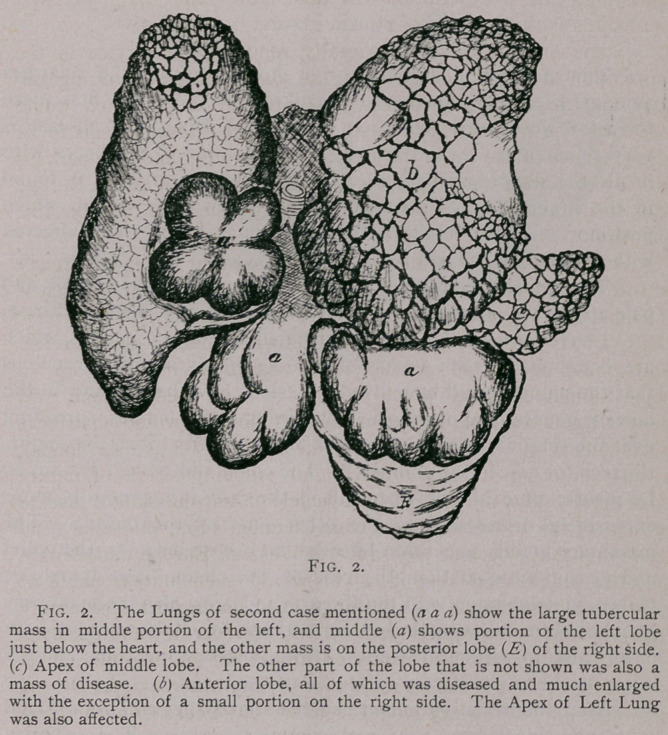


**Fig. 3. f3:**
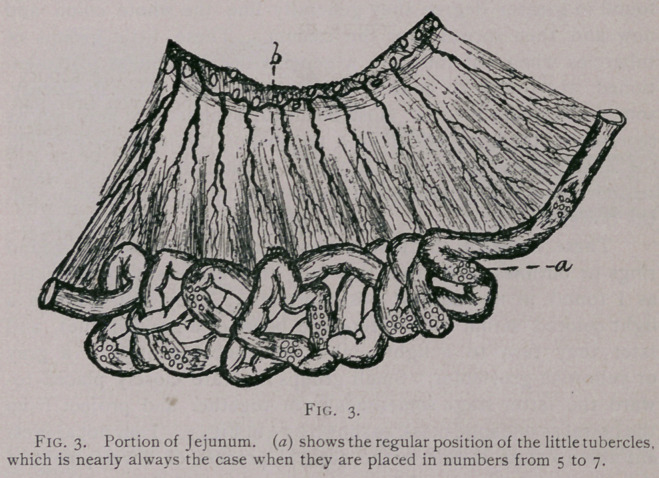


**Fig. 4. f4:**
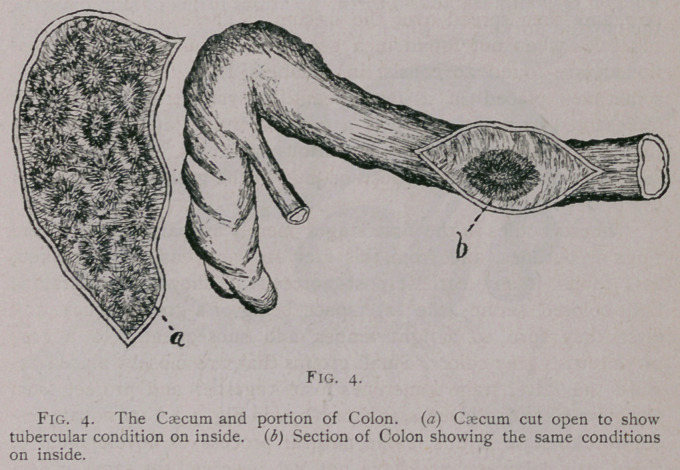


**Fig. 5. f5:**
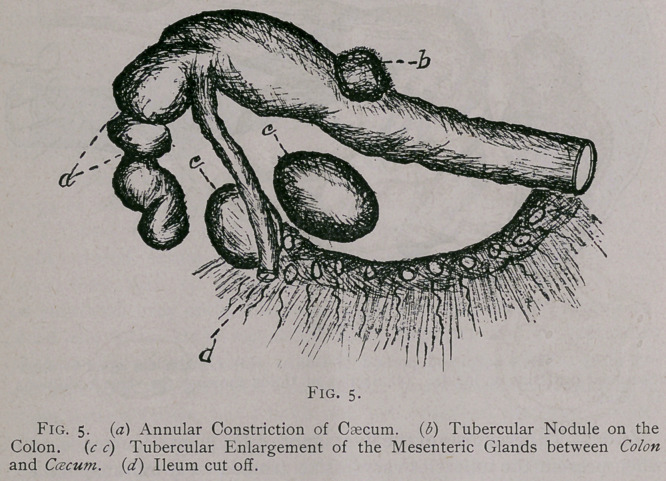


**Fig. 6. f6:**
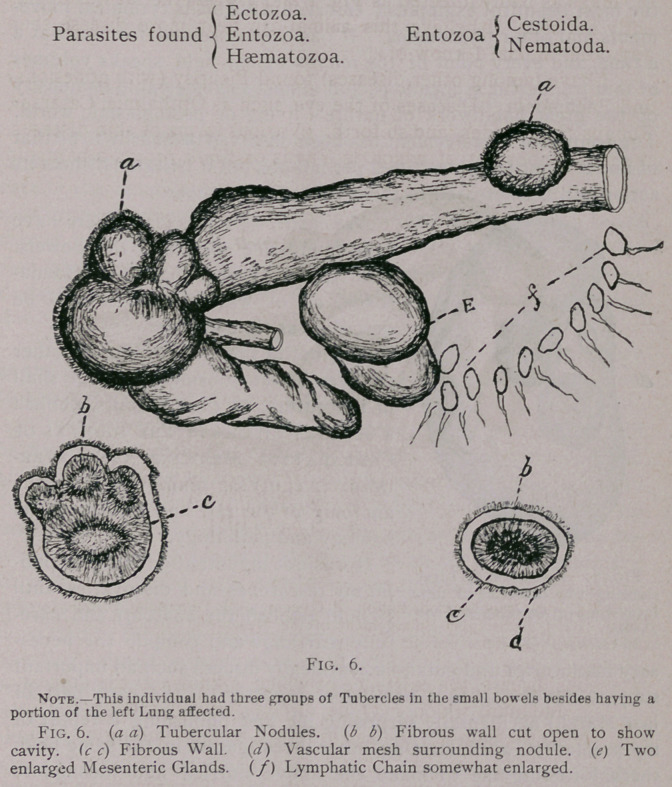


**Fig. 7. f7:**